# Hexa­kis­[di­methyl­tin(IV) difluoride] potassium iodide, 6Me_2_SnF_2_·KI: linear rods of potassium iodide penetrating the pores in planar layers of di­methyl­tin(IV) difluoride

**DOI:** 10.1107/S2056989026000265

**Published:** 2026-01-16

**Authors:** Johanna Vages, Tobias Gieschen, Kornelius Neue, Hans Reuter

**Affiliations:** a Chemistry, Osnabrück University, Barabarstr. 7, 49069 Osnabrück, Germany; Vienna University of Technology, Austria

**Keywords:** crystal structure, supra­molecular assembly, layer structure, octa­hedral coordination, tessellation, host–guest compound

## Abstract

The hexa­gonal host–guest title compound, poly[hexa­kis­[[di­methyl­tin(IV)]-di-μ-fluorido] potassium iodide], {[Sn(CH_3_)_2_F_2_]_6_·KI}_*n*_ or (Me_2_SnF_2_)_6_·KI, represents a layer structure of distorted {Me_2_SnF_4/2_} octa­hedra corner-linked *via* μ_2_-bonding fluorine atoms. Distortion of the octa­hedra concerns not only bond lengths [*d*(Sn—C) = 2.089 (2) Å, *d*(Sn—F) = 2.077 (1)/2.080 (1), 2.252 (1)/2.266 (1) Å] but also bond angles [〈(C—Sn—C) = 162.2 (1)°, 〈(F—Sn—F) = 77.11 (7)–119.57 (5)°] giving rise to a irregular quadrilateral, pseudo-equatorial plane of fluorine atoms around the central tin atom. In the planar (001) layers, the octa­hedra are arranged according to a snub hexa­gonal tiling (sr{3,6}) resulting in small trigonal and larger hexa­gonal pores. The latter are occupied by potassium ions [*d*(F⋯K) = 2.702 (2) Å, 6×], which in turn form linear rods with iodine ions [*d*(K⋯I) = 3.6702 (2) Å, 2×) perpendicular to adjacent layers.

## Abstract 2

In the solid state, hexa­kis­[di­methyl­tin(IV) difluoride] potassium iodide, (Me_2_SnF_2_)_6_·KI, consists of planar layers of corner-linked {Me_2_SnF_4/2_} octa­hedra in a snub hexa­gonal tiling (sr{3,6}) arrangement, which leads to the formation of hexa­gonal pores in which potassium cations are located. The latter are linearly connected to iodide anions perpendicular to the layers.

## 1. Chemical context

Di­methyl­tin(IV) difluoride, Me_2_SnF_2_, takes up a special position among the diorganotin(IV) dihalides as it is the only one that forms a layered structure with octa­hedrally coordinated tin atoms connected to each other *via* μ_2_-coordinating halogen atoms (Schlemper & Hamilton, 1966 ▸). In addition, the planar layers have some unique features, such as linear fluorine bridges and tin atoms with site symmetry of 4/*mmm*, so that the methyl groups are also arranged exactly linearly but disordered. Despite these unusual structural properties, nothing is yet known about its potential supra­molecular properties, which is certainly also due to the fact that the compound is largely insoluble.
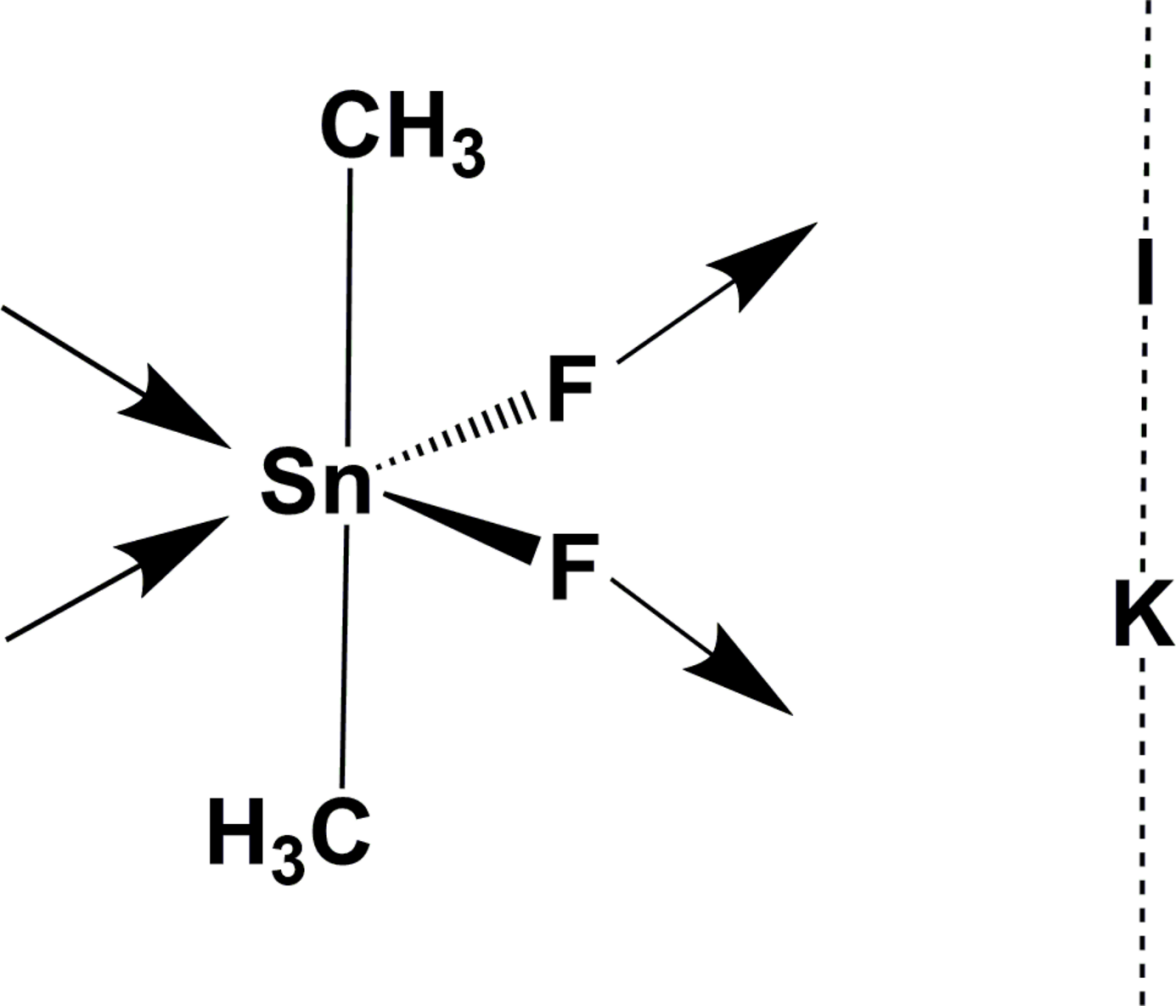


Like many other diorganotin(IV) difluorides, di­methyl­tin(IV) difluoride is most easily and cheaply prepared *via* a halide-exchange reaction of di­methyl­tin(IV) dichloride, Me_2_SnCl_2_, and potassium fluoride in ethanol or acetone as a solvent (Krause, 1918 ▸). By modifying these reaction conditions and using di­methyl­tin diiodide, Me_2_SnI_2_, instead of di­methyl­tin dichloride, it was possible for the first time to obtain not only the originally desired di­methyl­tin(IV) difluoride but also the host–guest compound of the *difluoride* with potassium iodide and the composition 6Me_2_SnF_2_·KI in a reproducible manner.

## 2. Structural commentary

**Figure 1:**
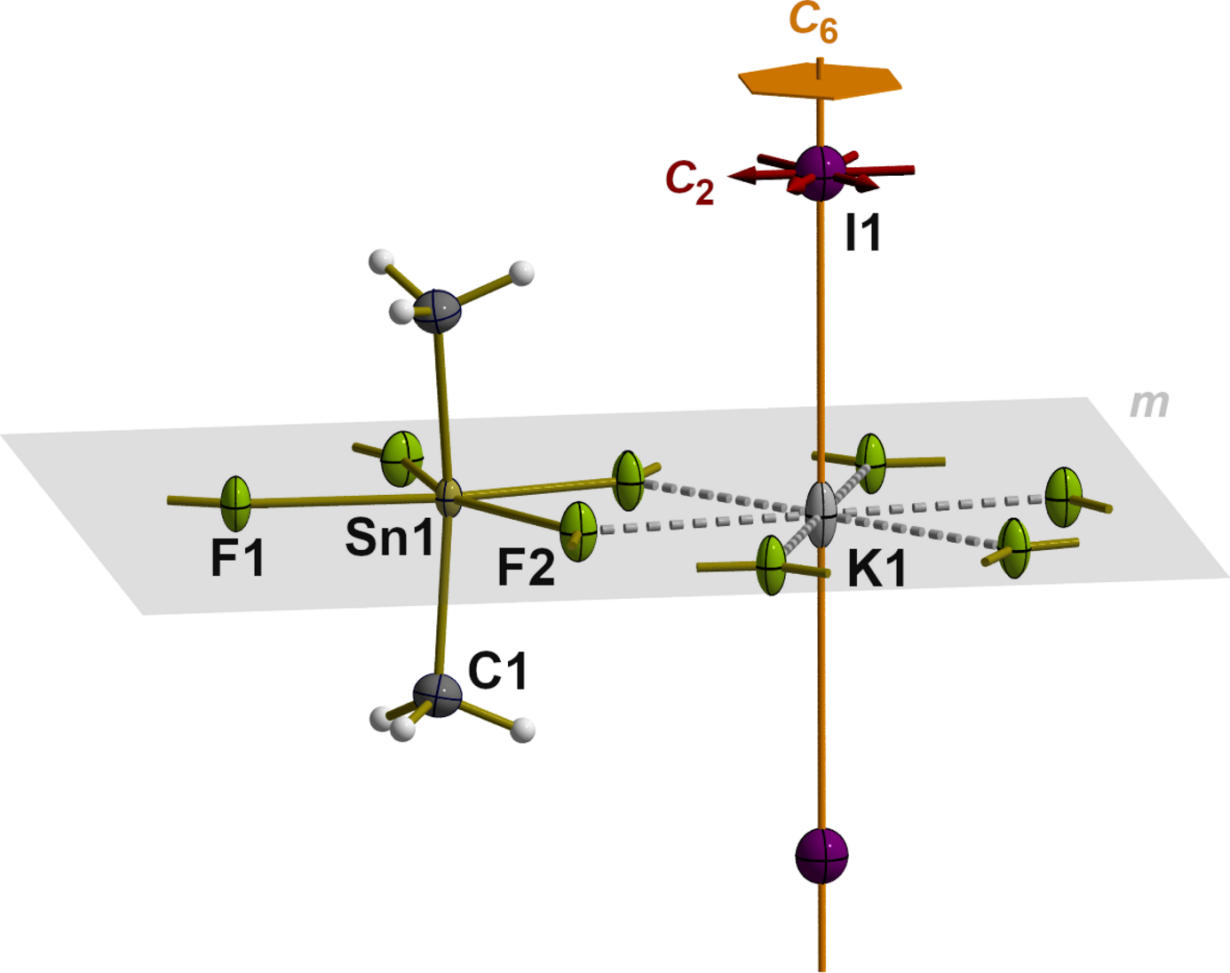
Ball-and-stick model showing the connectivity scheme between the atoms in (Me_2_SnF_2_)_6_·KI, the atom labeling of the asymmetric unit, and some symmetry elements (*m* = mirror plan, gray, *C*_2_ = twofold rotation axis, red arrow, *C*_6_ = sixfold rotation axis, orange hexa­gon). With exception of the hydrogen atoms, which are shown as spheres of arbitrary radius, all other atoms are drawn as displacement ellipsoids at the 70% probability level. Covalent bonds are drawn in orange–yellow, predominantly ionic fluorine–potassium inter­actions are visualized as dashed sticks in gray.

The title compound crystallizes in the hexa­gonal space group *P*6/*mcc* with two formula units in the unit cell. The asymmetric unit (Fig. 1 ▸) consists of one tin atom and two fluorine atoms all three lying on a crystallographic mirror plane (Wyckoff letter *l*) and the atoms of one methyl group in general position (Wyckoff letter *m*). In addition, the potassium cation occupies the special position of site symmetry 6/*m* (Wyckoff letter *b*) and the iodine atom the special position of site symmetry 622 (Wyckoff letter *a*). Overall, the combination of these building blocks results in a supra­molecular arrangement in which linear rods of potassium iodide penetrate the pores within planar layers of di­methyl­tin(IV) difluoride.

**Figure 2:**
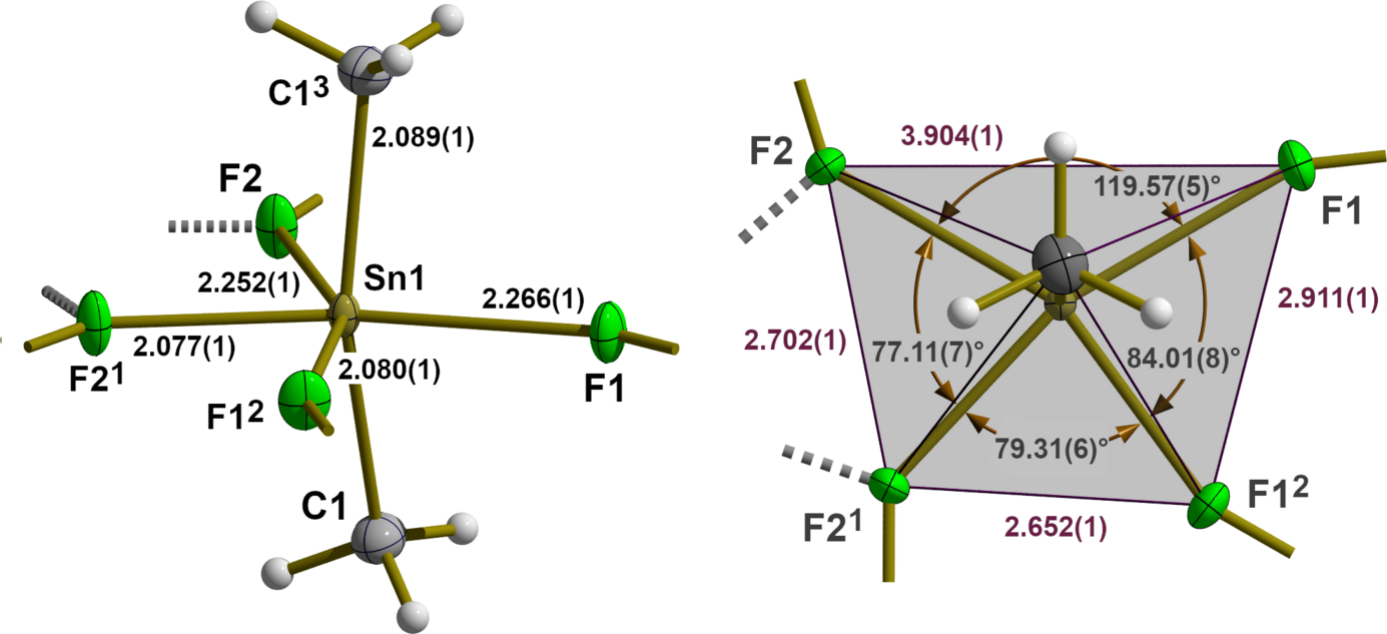
Ball-and-stick model with bond lengths and angles showing the octa­hedral coordination of the tin(IV) atom in side view (left) and top view (right). Inter­atomic fluorine⋯fluorine distances in the tin–fluorine plane (gray) are visualized on the right. [Symmetry codes used to generate equivalent atoms: (1) *y* − 1, −*x* + *y*, −*z* + 2; (2) −*y* + 1, *x* − *y* + 1, *z*; (3) *x*, *y*, −*z* + 2.]

The di­methyl­tin difluoride units of the title compound form exactly planar layers, as in Me_2_SnF_2_ itself (Schlemper & Hamilton, 1966 ▸). In contrast to the latter, the octa­hedral coordination of the tin(IV) atoms of the title compound, however, is much more distorted and the fluorine bridges are bent. Distortion of the {Me_2_SnF_4/2_} octa­hedron (Fig. 2 ▸) not only results from four different Sn—F distances but also from bond angles strongly deviating from 90° (Table 1 ▸). The Sn—F distances differ considerably and fall into two categories: two are very short (≃ 2.078 Å) and two are much longer (≃ 2.259 Å). In the case of Me_2_SnF_2_, all four Sn—F distances are the same [2.120 (5) Å]. Angular distortions in the {Me_2_SnF_4/2_} octa­hedron of the title compound are considerable, in particular within the tin-fluorine plane. On the one hand, there are angles that are significantly smaller [77.11 (7), 79.31 (6), 84.01 (8)°] than 90°, while one angle is significantly larger [119.57 (5)°] so that the exactly planar pseudo-equatorial plane takes the shape of an irregular quadrilateral [*d*(F⋯F) = 2.6524 (1), 2.7017 (1), 2.9112 (1), 3.9043 (1) Å]. The small angles result in very short fluorine–fluorine distances, which leads to a significant inter-penetration of the van der Waals spheres [*r*_vdW_(F) = 1.47 Å; Mantina *et al.*, 2009 ▸] of the corresponding fluorine atoms. Most remarkable, however, are the bond angles between *trans*-positioned atoms that increase to around 156° (Table 1 ▸).

So far, octa­hedral {Me_2_SnF_4_} building units have been found not only in Me_2_SnF_2_ (Schlemper & Hamilton, 1966 ▸) but also in the fluorido­stannates(IV) [Et_4_N][Me_4_Sn_3_F_5_] (Lambertsen *et al.*, 1992 ▸) and K_2_[Me_2_SnF_4_]·2H_2_O (Ahmed *et al.*, 2002 ▸). In the first, the two crystallographically independent building units are involved in the formation of bands whereby two fluorine atoms occupy terminal positions [*d*(Sn—F) = 2.026 (3) Å] and two bridging functions [*d*(Sn—F) = 2.115 (3)–2.272 (4) Å, 〈(Sn—F—Sn) = 150.1 (2)°/151.6 (2)°]; Sn—C distances are 2.105/2.117 Å and thus are somewhat longer than in the title compound (Table 1 ▸). The bond angles between *trans*-positioned ligands are all 180° in case of one tin atom and 167.0 (3)° between the carbon atoms and 175.7 (1)° between the fluorine atoms in the second tin atom. The pseudo-equatorial tin–fluorine planes are planar in both fluorido­stannates(IV) but more symmetrical than in the title compound. In [Et_4_N][Me_4_Sn_3_F_5_] (Lambertsen *et al.*, 1992 ▸), composed of two crystallographically independent tin atoms, one plane is rectangular [*d*(F⋯F) = 3.027 (6), 3.002 (5) Å], and the other is trapezoid [3.091 (6)/2.969 (5), 3.044 (4)/3.044 (4) Å]. In K_2_[Me_2_SnF_4_]·2H_2_O (Ahmed *et al.* 2002 ▸) the plane is rectangular [*d*(F⋯F) = 2.958 (14), 3.012 (13) Å], too.

Both fluorine atoms in 6Me_2_SnF_2_·KI connect two tin atoms in a bent μ_2_ coordination mode. In addition, the fluorine atom F2 is in contact with the potassium ion, but this contact [*d*(F⋯K) = 2.702 (1) Å] has no influence on the tin–fluorine distances, one of which is short and the other long. Only the bridging angle between the two tin atoms is reduced from 155.99 (8)° at F1 to 137.11 (7)° at F2 due to this contact.

**Figure 3:**
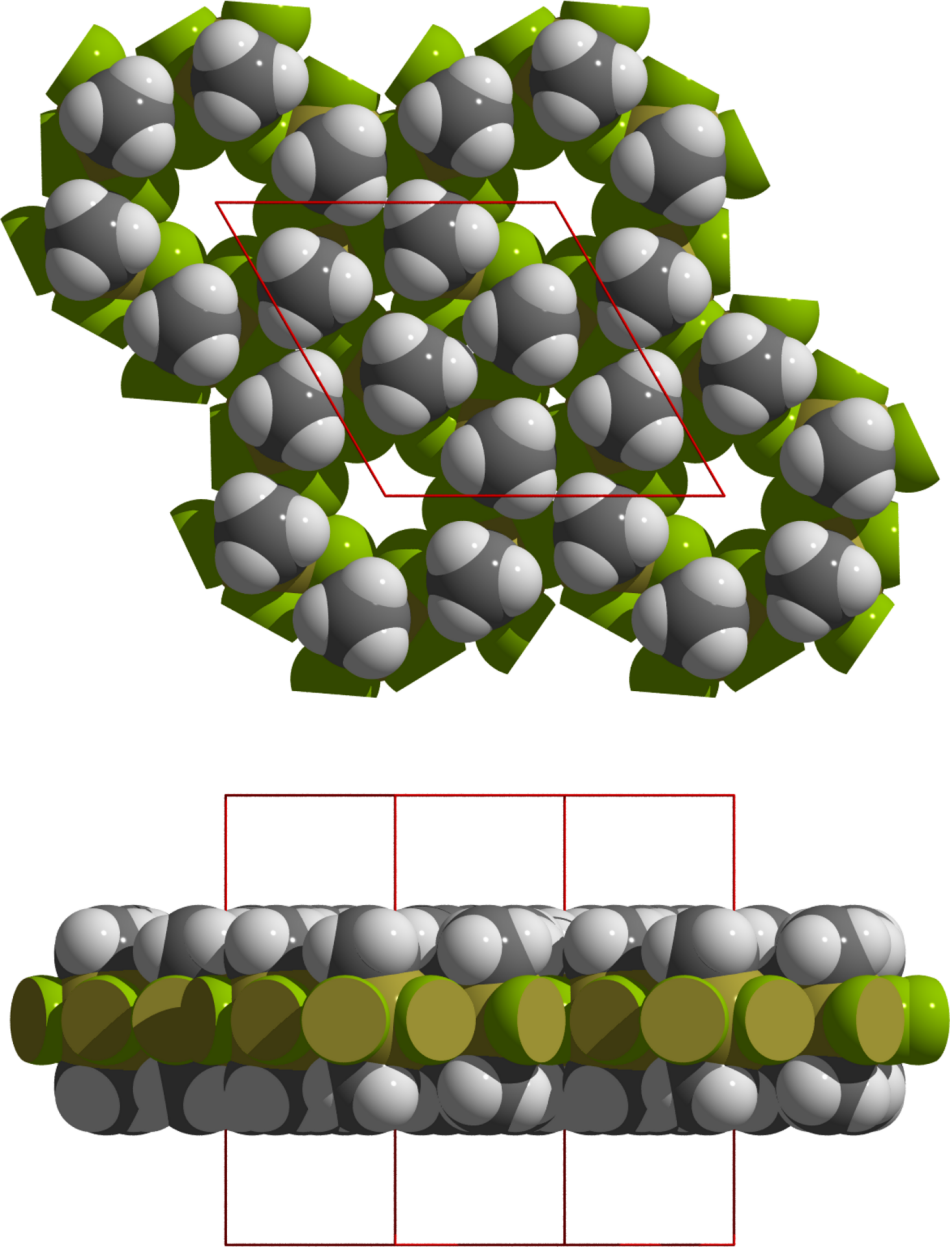
Space filling model showing the construction principle of a Me_2_SnF_2_ layer in relation to the unit cell (red) in top view (above) and side view (below). Atoms are visualized as single-colored or truncated, two-colored spheres according to their van der Waals radii and cut-offs based on the inter­section of the two spheres with cut-off faces showing the color of the inter­penetrating atom. Color code/van der Waals radii used: Sn = orange–yellow/2.17 Å, F = green/1.47 Å, C = dark gray/1.70 Å, H = white/1.1 Å.

**Figure 4:**
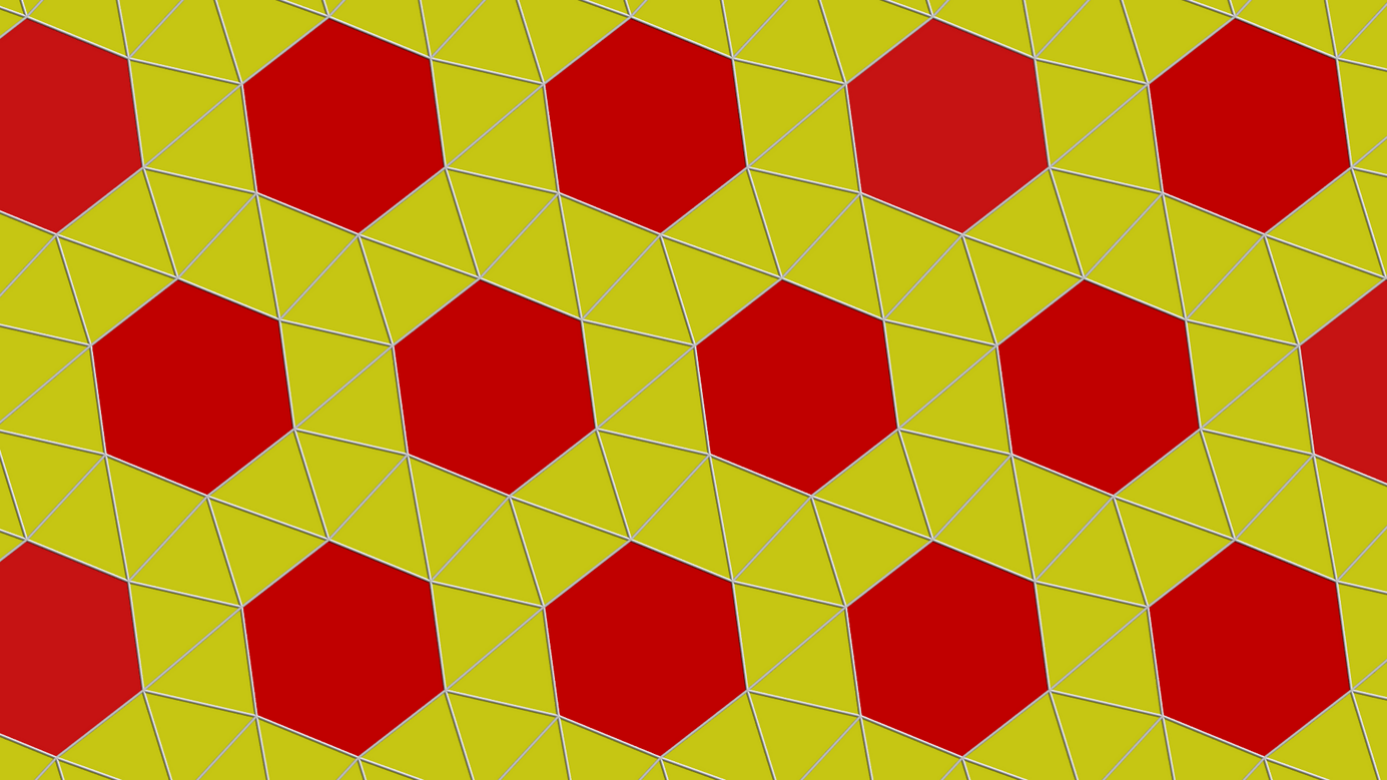
Details of the tessellation pattern in the Me_2_SnF_2_ layers of the title compound resulting from the positions of the tin atoms positioned in the corners of the polygons.

**Figure 5:**
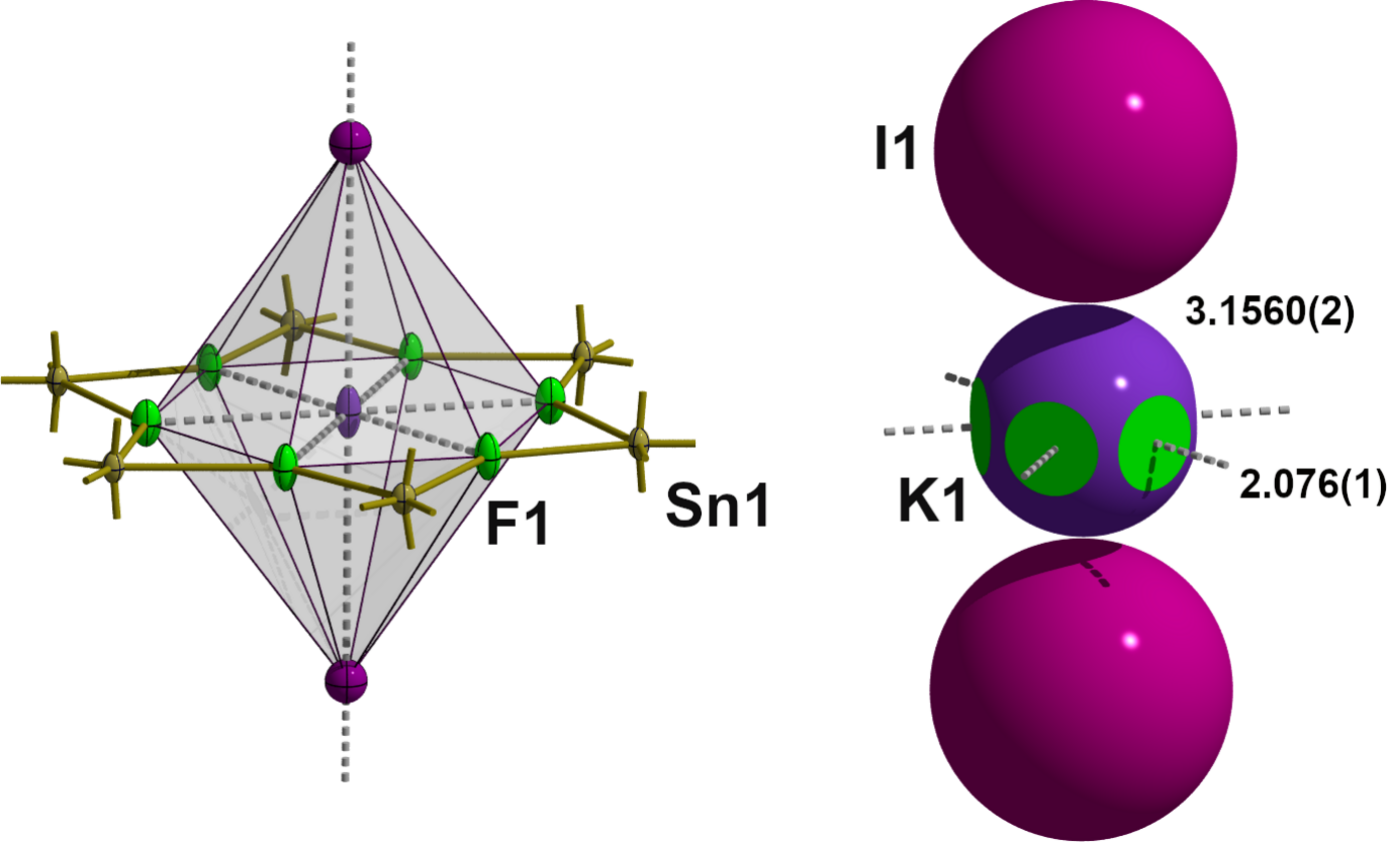
Ball-and-stick model (left) and space-filling model (right) of hexa­gonal–bipyramidal coordination of the potassium ion with K—F and K—I atom distances. In the space-filling model, atoms are visualized as single-colored or truncated, two-colored spheres according to their van der Waals radii and cut-offs based on the inter­section of the two spheres with cut-off faces showing the color of the inter­penetrating atom. Color code/van der Waals radii used: K = blue/2.17 Å, F = green/1.47 Å, I = violet/2.06 Å.

The bridging of the tin atoms by the fluorine atoms leads to a layered arrangement of the di­methyl­tin(IV) difluoride building blocks, whereby the symmetrically related methyl groups [*d*(Sn—C) = 2.089 (2) Å] are almost perpendicular to the exactly planar tin–fluorine plane (Fig. 3 ▸). In the layers, the tin atoms are arranged in such a way that slightly distorted triangles [*d*(Sn⋯Sn) = 4.0298 (1)/4.2507 (2)/4.7220 (1) Å] and regular hexa­gons form a semi-regular 3-3-3-3-6 tessellation (Fig. 4 ▸). On each vertex of this snub hexa­gonal tiling (Schläfli-symbol sr{3,6}), there are four triangles and one hexa­gon. While the bridging fluorine atoms fill the space in the triangles practically seamlessly, this is not the case in the hexa­gons. The resulting pores [*d*(F⋯F) = 5.403 (1) Å] are large enough to incorporate potassium cations. As a result of the special position of the potassium cation, it is hexa­gonal–bipyramidally coordinated by six equatorially bound fluorine atoms [*d*(K⋯F) = 2.702 (2) Å] and two axially bound iodine anions [*d*(K⋯I) = 3.6702 (2) Å] (Fig. 5 ▸), resulting in linear rods of potassium iodine extending along [001]. In potassium fluoride, KF, and potassium iodide, KI, the potassium atoms are octa­hedrally coordinated (both adopt the NaCl structure type), and the corresponding potassium–halide distances are *d*(K⋯F) = 2.672 (3) Å (*a* = 5.334 (3) Å, *T* = 295 (2) K; Broch *et al.*, 1929 ▸), and *d*(K⋯I) = 3.529 Å (*a* = 7.059 Å, *T* = 295 (2) K; Teatum & Smith, 1957 ▸) and 3.5328 (2) Å (*a* = 7.0655 (2), *T* = 295 (2) K; Hambling, 1953 ▸), respectively, indicating predominantly ionic bonding within the rods and between the potassium cations and the fluorine atoms of the tin–fluorine layers. Calculations of the bond lengths based on the ionic radii lead to similar results, *i.e*. K⋯I distances are even slightly shorter (3.58 Å with *r*_K_[6] = 1.52 Å, *r*_I_[6] = 2.06 Å; Shannon, 1976 ▸). In the fluorido­stannate(IV) K_2_[Me_2_SnF_4_]·2H_2_O the K⋯F contacts are about 0.1 Å shorter [2.595 (12), 2.617 (2) Å].

**Figure 6:**
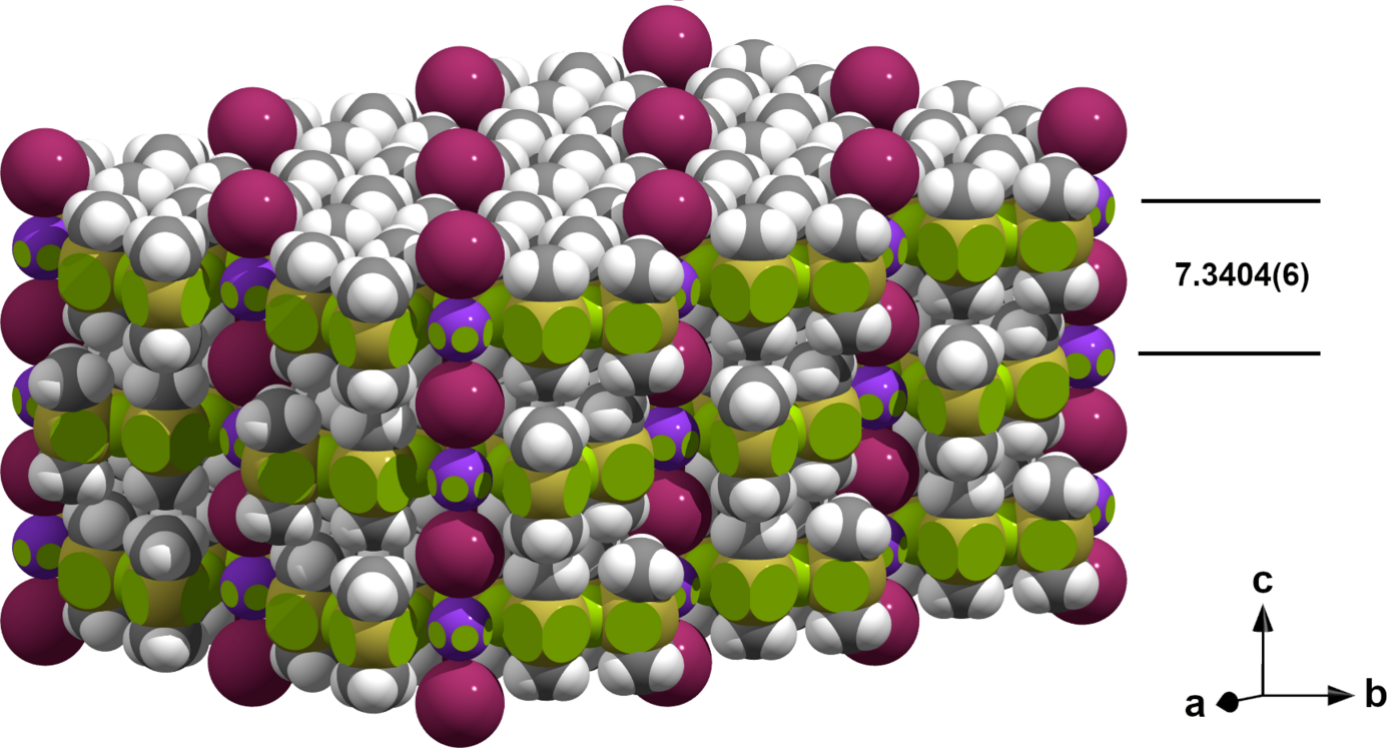
Space-filling model (3 × 2 × 1 unit cells) based on the van der Waals radii of tin (magenta, 2.17 Å), fluorine (green, 1.47 Å), carbon (dark gray, 1.70 Å), hydrogen (white, 1.10 Å) and ionic radii of potassium (blue, 1.52 Å), iodine (violet, 2.06 Å) that describes the packing of the Me_2_SnF_2_ layers; layer spacing in Å.

The distances between the Sn—F planes are *c*/2 = 7.3404 (6) Å (Fig. 6 ▸), while they are somewhat smaller [7.08 (2) Å] in Me_2_SnF_2_. The slightly longer distance in the title compound is due to the fact that the methyl groups of two neighboring layers are directly opposite each other, while they are laterally offset in the guest-free *difluoride*. There are no inter­actions of the iodine anions except with the potassium cations. They are, however, regularly surrounded in a hexa­gonal prismatic shape from six symmetry-equivalent hydrogen atoms [H13] in a distance of 3.446 Å that is significant longer than the sum (3.08 Å) of the van der Waals radii (Mantina *et al.* 2009 ▸) of iodine (1.98 Å) and hydrogen (1.10 Å).

## 3. Synthesis and crystallization

0.80 g (1.99 mmol) of Me_2_SnI_2_ were dissolved at room temperature in 20 ml of ethanol to which a solution of 0.10 g (2.08 mmol) KF in 10 ml of water was added while stirring. The colorless, voluminous precipitate of Me_2_SnF_2_ that formed immediately was filtered off after 20 min and washed twice with 5 ml toluene, yield: 0.24 g (1.29 mmol, 65%). After a few days during which a large part of the solvents had evaporated, the host–guest compound 6Me_2_SnF_2_·KI crystallized out of the remaining reaction solution, yield: 55 mg.

**Figure 7:**
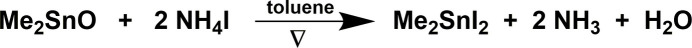
Reaction equation for the formation of Me_2_SnI_2_.

Di­methyl­tin(IV) diiodide, Me_2_SnI_2_, was prepared from di­methyl­tin(IV) oxide, Me_2_SnO, and ammonium iodide, NH_4_I, in a molar ratio of 1:2 *via* the release of water and ammonia (Fig. 7 ▸). For this purpose, 2.97 g (18 mmol) Me_2_SnO and 6.26 g (43.2 mmol) NH_4_I were suspended in 200 ml toluene and the mixture heated to boiling under reflux in a soxhlet apparatus. The water formed during the reaction was removed using silica gel in an extraction sleeve. After 8 h, the mixture was filtered off hot and most of the solvent was distilled off. During the evaporation of the remaining solvent, di­methyl­tin(IV) diiodide crystallized out as very large, dark-yellow crystals over the course of 2 d. Yield: 4.13 g (10.26 mmol; 57%). ^1^H NMR (250 MHz, CDCl_3_): δ, ^*n*^*J*(^119/117^Sn–^1^H) (ppm, Hz) 1.57,62 (*s*, CH_3_); ^13^C NMR (250 MHz, CDCl_3_): δ,^*n*^J(^119/117^Sn–^13^C) (ppm, Hz) 6.02, 387.3/370.2 (CH_3_)/^1^J); analysis: calculated for C_2_H_6_I_2_Sn (402.59): C 5.97, H 1.50, found C 5.99, H 1.48%.

## 4. Refinement details

Crystal data, data collection and structure refinement details are summarized in Table 2 ▸. Methyl H atoms were placed geometrically and allowed to ride on the C atom (AFIX 137; Sheldrick, 2015 ▸) with *d*(C—H) = 0.98 Å) and a common *U*_iso_(H) parameter.

## Supplementary Material

Crystal structure: contains datablock(s) I. DOI: 10.1107/S2056989026000265/wm5784sup1.cif

Structure factors: contains datablock(s) I. DOI: 10.1107/S2056989026000265/wm5784Isup2.hkl

CCDC reference: 2521792

Additional supporting information:  crystallographic information; 3D view; checkCIF report

## Figures and Tables

**Table 1:** Selected geometric parameters (Å, °)

Sn1—F1	2.266 (1)	Sn1—F1^ii^	2.080 (1)
Sn1—F2^i^	2.077 (1)	Sn1—C1^iii^	2.089 (2)
Sn1—F2	2.252 (1)	Sn1—C1	2.089 (2)
			
C1^iii^—Sn1—C1	162.2 (1)	Sn1^iv^—F1—Sn1	155.99 (8)
F1^ii^—Sn1—F2	156.42 (5)	Sn1^v^—F2—Sn1	137.11 (7)

Symmetry codes: (i) 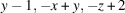; (ii) 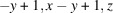; (iii) 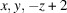; (iv) 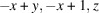; (v) 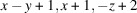.

**Table 2:** Experimental details

Crystal data	
Chemical formula	[Sn(CH_3_)_2_F_2_]_6_·KI
*M*_r_	1286.55
Crystal system, space group	Hexagonal, *P*6/*mcc*
Temperature (K)	100
*a*, *c* (Å)	11.0616 (4), 14.6807 (6)
*V* (Å^3^)	1555.65 (13)
*Z*	2
Radiation type	Mo *K*α
μ (mm^−1^)	5.94
Crystal size (mm)	0.22 × 0.14 × 0.05

Data collection	
Diffractometer	Bruker APEXII CCD
Absorption correction	Multi-scan (*SADABS*; Krause *et al.*, 2015 ▸)
*T*_min_, *T*_max_	0.451, 0.712
No. of measured, independent and observed [*I* > 2σ(*I*)] reflections	51729, 662, 635
*R*_int_	0.080
(sin θ/λ)_max_ (Å^−1^)	0.660

Refinement	
*R*[*F*^2^ > 2σ(*F*^2^)], *wR*(*F*^2^), *S*	0.013, 0.028, 1.17
No. of reflections	662
No. of parameters	35
H-atom treatment	H-atom parameters constrained
Δρ_max_, Δρ_min_ (e Å^−3^)	0.65, −0.40

Computer programs: *APEX2* and *SAINT* (Bruker, 2009 ▸), *SHELXS* (Sheldrick 2008 ▸), *SHELXL* (Sheldrick, 2015 ▸), *DIAMOND* (Brandenburg, 2006 ▸), *Mercury* (Macrae *et al.* (2020 ▸) and *publCIF* (Westrip, 2010 ▸).

## supplementary crystallographic information

### Poly[hexakis[[dimethyltin(IV)]-di-µ-fluorido] potassium iodide] Crystal data

**Table:**

[Sn(CH_3_)_2_F_2_]_6_·KI	*D*_x_ = 2.747 Mg m^−^^3^
*M**_r_* = 1286.55	Mo *K*α radiation, λ = 0.71073 Å
Hexagonal, *P*6/*m**c**c*	Cell parameters from 9284 reflections
*a* = 11.0616 (4) Å	θ = 2.8–29.1°
*c* = 14.6807 (6) Å	µ = 5.94 mm^−^^1^
*V* = 1555.65 (13) Å^3^	*T* = 100 K
*Z* = 2	Plate, colourless
*F*(000) = 1176	0.22 × 0.14 × 0.05 mm

### Poly[hexakis[[dimethyltin(IV)]-di-µ-fluorido] potassium iodide] Data collection

**Table:**

Bruker APEXII CCD diffractometer	635 reflections with *I* > 2σ(*I*)
φ and ω scans	*R*_int_ = 0.080
Absorption correction: multi-scan (SADABS; Krause *et al.*, 2015)	θ_max_ = 28.0°, θ_min_ = 2.8°
*T*_min_ = 0.451, *T*_max_ = 0.712	*h* = −14→14
51729 measured reflections	*k* = −14→14
662 independent reflections	*l* = −19→19

### Poly[hexakis[[dimethyltin(IV)]-di-µ-fluorido] potassium iodide] Refinement

**Table:**

Refinement on *F*^2^	Hydrogen site location: inferred from neighbouring sites
Least-squares matrix: full	H-atom parameters constrained
*R*[*F*^2^ > 2σ(*F*^2^)] = 0.013	*w* = 1/[σ^2^(*F*_o_^2^) + (0.0086*P*)^2^ + 1.4878*P*] where *P* = (*F*_o_^2^ + 2*F*_c_^2^)/3
*wR*(*F*^2^) = 0.028	(Δ/σ)_max_ = 0.001
*S* = 1.17	Δρ_max_ = 0.65 e Å^−^^3^
662 reflections	Δρ_min_ = −0.39 e Å^−^^3^
35 parameters	Extinction correction: SHELXL (Sheldrick, 2015), Fc^*^=kFc[1+0.001xFc^2^λ^3^/sin(2θ)]^-1/4^
0 restraints	Extinction coefficient: 0.00069 (7)
Primary atom site location: structure-invariant direct methods	

### Poly[hexakis[[dimethyltin(IV)]-di-µ-fluorido] potassium iodide] Special details

**Table:**

Geometry. All esds (except the esd in the dihedral angle between two l.s. planes) are estimated using the full covariance matrix. The cell esds are taken into account individually in the estimation of esds in distances, angles and torsion angles; correlations between esds in cell parameters are only used when they are defined by crystal symmetry. An approximate (isotropic) treatment of cell esds is used for estimating esds involving l.s. planes.

### Poly[hexakis[[dimethyltin(IV)]-di-µ-fluorido] potassium iodide] Fractional atomic coordinates and isotropic or equivalent isotropic displacement parameters (Å^2^)

**Table:**

	*x*	*y*	*z*	*U*_iso_*/*U*_eq_	
Sn1	0.25750 (2)	0.84066 (2)	1.0000	0.00817 (7)	
F1	0.45960 (15)	0.83530 (15)	1.0000	0.0150 (3)	
F2	0.26458 (14)	1.04772 (14)	1.0000	0.0145 (3)	
C1	0.2871 (2)	0.8695 (2)	0.85944 (14)	0.0177 (4)	
H11	0.2895	0.7897	0.8325	0.044 (5)*	
H12	0.3755	0.9552	0.8472	0.044 (5)*	
H13	0.2101	0.8772	0.8326	0.044 (5)*	
K1	0.0000	1.0000	1.0000	0.0162 (3)	
I1	0.0000	1.0000	1.2500	0.02125 (11)	

### Poly[hexakis[[dimethyltin(IV)]-di-µ-fluorido] potassium iodide] Atomic displacement parameters (Å^2^)

**Table:**

	*U*^11^	*U*^22^	*U*^33^	*U*^12^	*U*^13^	*U*^23^
Sn1	0.00640 (10)	0.00645 (9)	0.01217 (10)	0.00359 (7)	0.000	0.000
F1	0.0102 (7)	0.0154 (8)	0.0228 (8)	0.0091 (6)	0.000	0.000
F2	0.0094 (7)	0.0068 (6)	0.0266 (8)	0.0035 (6)	0.000	0.000
C1	0.0192 (9)	0.0217 (9)	0.0142 (9)	0.0116 (8)	0.0012 (7)	0.0010 (7)
K1	0.0073 (4)	0.0073 (4)	0.0338 (7)	0.00366 (18)	0.000	0.000
I1	0.02021 (14)	0.02021 (14)	0.0233 (2)	0.01011 (7)	0.000	0.000

### Poly[hexakis[[dimethyltin(IV)]-di-µ-fluorido] potassium iodide] Geometric parameters (Å, º)

**Table:**

Sn1—F1	2.266 (1)	K1—F2^vi^	2.702 (1)
Sn1—F2^i^	2.077 (1)	K1—F2^vii^	2.702 (1)
Sn1—F2	2.252 (1)	K1—F2^i^	2.702 (1)
Sn1—F1^ii^	2.080 (1)	K1—I1	3.6702 (2)
Sn1—C1^iii^	2.089 (2)	K1—I1^vi^	3.6702 (2)
Sn1—C1	2.089 (2)	C1—H11	0.9800
K1—F2	2.702 (1)	C1—H12	0.9800
K1—F2^iv^	2.702 (1)	C1—H13	0.9800
K1—F2^v^	2.702 (1)		
			
F2^i^—Sn1—F1^ii^	79.31 (6)	F2^vi^—K1—F2^i^	120.000 (1)
F2^i^—Sn1—C1^iii^	96.47 (5)	F2^vii^—K1—F2^i^	180.0
F1^ii^—Sn1—C1^iii^	97.19 (5)	F2^iv^—K1—F2	120.0
F2^i^—Sn1—C1	96.46 (5)	F2^v^—K1—F2	60.0
F1^ii^—Sn1—C1	97.19 (5)	F2^vi^—K1—F2	180.0
C1^iii^—Sn1—C1	162.2 (1)	F2^vii^—K1—F2	120.0
F2^i^—Sn1—F2	77.11 (7)	F2^i^—K1—F2	60.000 (1)
F1^ii^—Sn1—F2	156.42 (5)	F2^iv^—K1—I1	90.0
C1^iii^—Sn1—F2	85.50 (5)	F2^v^—K1—I1	90.0
C1—Sn1—F2	85.50 (5)	F2^vi^—K1—I1	90.0
F2^i^—Sn1—F1	163.31 (5)	F2^vii^—K1—I1	90.0
F1^ii^—Sn1—F1	84.01 (8)	F2^i^—K1—I1	90.0
C1^iii^—Sn1—F1	85.56 (5)	F2—K1—I1	90.0
C1—Sn1—F1	85.56 (5)	F2^iv^—K1—I1^vi^	90.0
F2—Sn1—F1	119.57 (5)	F2^v^—K1—I1^vi^	90.0
Sn1^viii^—F1—Sn1	155.99 (8)	F2^vi^—K1—I1^vi^	90.0
Sn1^v^—F2—Sn1	137.11 (7)	F2^vii^—K1—I1^vi^	90.0
Sn1^v^—F2—K1	114.35 (6)	F2^i^—K1—I1^vi^	90.0
Sn1—F2—K1	108.53 (5)	F2—K1—I1^vi^	90.0
F2^iv^—K1—F2^v^	180.0	I1—K1—I1^vi^	180.0
F2^iv^—K1—F2^vi^	60.0	K1^ix^—I1—K1	180.0
F2^v^—K1—F2^vi^	120.0	Sn1—C1—H11	109.5
F2^iv^—K1—F2^vii^	120.000 (1)	Sn1—C1—H12	109.5
F2^v^—K1—F2^vii^	60.0	H11—C1—H12	109.5
F2^vi^—K1—F2^vii^	60.0	Sn1—C1—H13	109.5
F2^iv^—K1—F2^i^	60.0	H11—C1—H13	109.5
F2^v^—K1—F2^i^	120.000 (1)	H12—C1—H13	109.5

Symmetry codes: (i) *y*−1, −*x*+*y*, −*z*+2; (ii) −*y*+1, *x*−*y*+1, *z*; (iii) *x*, *y*, −*z*+2; (iv) −*x*+*y*−1, −*x*+1, *z*; (v) *x*−*y*+1, *x*+1, −*z*+2; (vi) −*x*, −*y*+2, −*z*+2; (vii) −*y*+1, *x*−*y*+2, *z*; (viii) −*x*+*y*, −*x*+1, *z*; (ix) *y*−1, *x*+1, −*z*+5/2.
